# Cnidarians as a Source of New Marine Bioactive Compounds—An Overview of the Last Decade and Future Steps for Bioprospecting

**DOI:** 10.3390/md9101860

**Published:** 2011-10-10

**Authors:** Joana Rocha, Luisa Peixe, Newton C.M. Gomes, Ricardo Calado

**Affiliations:** 1 Instituto de Ciencias Biomedicas Abel Salazar, Universidade do Porto, Largo Professor Abel Salazar no. 2, 4099-003 Porto, Portugal; 2 Departmento de Biologia & CESAM, Universidade de Aveiro, Campus Universitario de Santiago, 3810-193 Aveiro, Portugal; E-Mail: gomesncm@ua.pt; 3 REQUIMTE, Laboratorio de Microbiologia, Faculdade de Farmacia, Universidade do Porto, Rua Anibal Cunha no. 164, 4050-047 Porto, Portugal; E-Mail: lpeixe@ff.up.pt

**Keywords:** coral, sea fan, sea anemone, biotechnology

## Abstract

Marine invertebrates are rich sources of bioactive compounds and their biotechnological potential attracts scientific and economic interest worldwide. Although sponges are the foremost providers of marine bioactive compounds, cnidarians are also being studied with promising results. This diverse group of marine invertebrates includes over 11,000 species, 7500 of them belonging to the class Anthozoa. We present an overview of some of the most promising marine bioactive compounds from a therapeutic point of view isolated from cnidarians in the first decade of the 21st century. Anthozoan orders Alcyonacea and Gorgonacea exhibit by far the highest number of species yielding promising compounds. Antitumor activity has been the major area of interest in the screening of cnidarian compounds, the most promising ones being terpenoids (monoterpenoids, diterpenoids, sesquiterpenoids). We also discuss the future of bioprospecting for new marine bioactive compounds produced by cnidarians.

## 1. Introduction

In terms of biodiversity, marine environments are among the richest and most complex ecosystems. Harsh chemical and physical conditions in the environment have been important drivers for the production of a variety of molecules with unique structural features. These marine molecules exhibit various types of biological activities [1], with compounds of high economic interest having potential applications in the pharmaceutical and medical sectors. Although nearly 20,000 compounds have been discovered since the field of marine bioactive compound biochemistry began in the mid-1960s, only a very limited number have seen industrial application. It has been clear since marine bioprospecting began that the world’s oceans and their diverse biota represent a significant resource, perhaps the greatest resource on Earth, for the discovery of new bioactive compounds. Early National Cancer Institute (NCI) programs in the USA demonstrated that marine invertebrates were a superb source of potential lead molecules. The decisive boost to this new age of bioprospecting was provided by the NCI when it was found that bioassays with marine organism extracts were far more likely to yield anticancer drugs than terrestrial sources [2]. In this way, it is not surprising that over the past 40 years major advances in the discovery of marine drugs have been recorded in clinical trials for cancer [3]. Apart from anticancer activity, these compounds have proven to be an abundant source of pharmacologically active agents for the production of therapeutic entities [4] against AIDS, inflammatory conditions and microbial diseases.

Marine bioactive compounds display varied potential applications, namely as molecular tools, in cosmetics, as fine chemicals, as nutraceuticals and in agrochemical industries [5]. Although only a few marine-derived products are currently on the market (e.g., Prialt^®^ and Yondelis^®^), several new compounds are now in the clinical pipeline and several more are in clinical development. The few approvals so far for the commercialization of drugs from the sea have not been due to a lack of discovery of novel marine bioactive compounds, but because of the complexity of issues raised upon the development of these products [4]. Faulkner [6–20], Blunt *et al*. [21–29], and Mayer [30–38] have provided extensive reviews on the total number of marine natural products (MNPs) discovered over the last 25 years, the most promising ones being produced by marine invertebrates. Sponges (phylum Porifera) have long been recognized as the most interesting group of marine invertebrates for the discovery of new drugs [5,39,40]. However, with growing bioprospecting efforts and the screening of previously unexplored marine habitats, the biotechnological potential of other groups of marine invertebrates has also started to attract the attention of researchers. The ability of cnidarians (such as jellyfish, sea anemones and corals) to produce powerful toxins and venoms [41] has been well documented. However, further research has demonstrated that MNPs produced by cnidarians are more than toxins and venoms. The phylum Cnidaria is a large, diverse and ecologically important group of marine invertebrates that includes over 11,000 extant species [42]. Over 3000 MNPs have been described from this phylum alone, mostly in the last decade.

In this work, we present an overview of the most promising marine bioactive compounds isolated from cnidarians in the first decade of the 21st century, which may have applications in the therapy of human diseases. The present study also discusses future perspectives for the bioprospecting of new MNPs produced by this speciose group of marine invertebrates.

## 2. Methodology

The most relevant peer reviewed literature published during the first decade of the 21st century covering MNPs was surveyed for the present work [18–37]. During this period alone, over 2000 molecules from cnidarians were described. In order to focus our study and address only those compounds displaying a high potential for industrial applications, we have decided to use as guidelines the values of IC_50_ (half maximal inhibitory concentration). IC_50_ is a quantitative measure which indicates how much of a particular substance (inhibitor) is needed to inhibit a given biological process or component of a process by half. It is important to highlight that the NCI has renamed the IC_50_ to GI_50_ [43] in order to emphasize the correction for cell count at time zero in cancer cells; in this way, some results on this quantitative measure are now also presented under these directives. Additionally, the ED_50_ (the median dose that produces the desired effect of a drug in half the test population) was also used to identify promising marine bioactive compounds produced by cnidarians. Only the compounds displaying an IC_50_ ≤ 10.0 μg/mL or μM (except where stated otherwise) and ED_50_ ≤ 4.0 μg/mL were considered for the present study, as these values are commonly used in the surveyed literature to ascertain relevant bioactivity (e.g., [44,45]). In the few cases were neither IC_50_ nor ED_50_ values were described for a MNP in a manuscript, that compound was selected to be part of the present survey only if either the authors of that manuscript, or those citing that manuscript, clearly stated that the results recorded were highly promising for industrial applications. All species producing the compounds selected for the present work were grouped into classes and orders of phylum Cnidaria (Table 1) (according to the classification proposed in the World Register of Marine Species (WoRMS)) [46].

This approach allowed us to identify which taxonomic groups of cnidarians screened so far display the highest potential to yield new drugs or pharmacological products derived from marine bioactive compounds. Nonetheless, it is important to highlight that cnidarian species identification is a challenging task and it is possible that some of the species (or even genera) referred to in the scientific literature may not be correct [47]. In this way, it is of paramount importance that in future works the authors addressing marine bioactive compounds produced by cnidarians provide a detailed description on how target species have been identified.

## 3. Class Anthozoa

Class Anthozoa currently includes 10 orders and over 7500 valid species (about 2/3 of all known cnidarian species) (Table 1). Within the Anthozoa, the order Alcyonacea (soft corals) and Gorgonacea (sea fans) are the ones which have contributed with the highest number of promising bioactive marine compounds, although other orders, such as Actiniaria (sea anemones) and Scleractinia (hard corals), have also yielded relevant compounds [48–51].

### 3.1. Order Alcyonacea (Soft Corals)

Soft corals are generally brightly colored and rich in nutritionally important substances. However, the incidence of predation in the majority of these organisms is low due to the toxic compounds they produce to deter predators [52]. Several biosynthetic studies have been carried out on the metabolites of soft corals [53] and some of those compounds have already shown to have great potential for the development of new pharmaceuticals and antifoulants. Table 2 summarizes the most promising compounds from order Alcyonacea (class Anthozoa) described in the present review.

**Figure 1:**
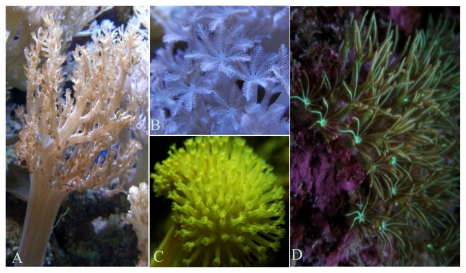
Some cnidarians addressed in this review (all images by Ricardo Calado). (**A**) *Sinularia* sp.; (**B**) *Xenia* sp.; (**C**) *Sarcophyton* sp.; (**D**) *Briareum* sp.

Soft corals are rich sources of secondary metabolites such as diterpenes, sesquiterpenes, furanoditerpenes, terpenoids, capnellenes and steroids (e.g., *Lobophytum*, *Sinularia* (Figure 1A), *Sarcophyton* [86] (Figure 1C), *Capnella* [87], *Dendronephthya* [78]), that have shown to display HIV-inhibitory [57], cytotoxic [88,89], anti-inflammatory [90,91], anticancer [92,93] and antimicrobial activity [94], as well as cardiac and vascular responses [95]. Soft corals of the family Nephtheidae are known for their content of sesquiterpenes and particularly capnellenes [28]. Some sesquiterpenes isolated from *Capnella imbricate* [87,96–98] showed anti-inflammatory activity and a dihydroxycapnellene (capnell-9(12)-ene-8β,10α-diol) from *Dendronephthya rubeola* demonstrated a good antiproliferative activity against murine fibroblasts cell line (L-929, GI_50_ 6.8 μM/L) and a good cytotoxicity against cancer cell lines implicated in human leukemia (K-562, IC_50_ 0.7 μM) and human cervix carcinoma (HeLa, IC_50_ 7.6 μM) [78]. Capnell-9(12)-ene-8β,10α-diol strongly inhibits the interaction of the oncogenic transcription factor Myc with its partner protein Max [79,80], making it a therapeutically interesting compound in oncology [78]. *Nephthea chabroli* also produces a nor-sisquiterpene compound, chabranol, which displays moderate cytotoxicity against P-388 (mouse lymphocytic leukemia cells) with an ED_50_ 1.81 μg/mL [81]. *Nephthea erecta* produces two proteins in mediated inflammatory responses, the oxygenated ergostanoids 1 and 3. These compounds at a concentration of 10 μM significantly reduced the levels of the iNOS (inducible nitric oxide synthase) (45.8 ± 9.9 and 33.6 ± 20.6%, respectively) and COX-2 (cyclooxygenase-2) protein (68.1 ± 2.3 and 10.3 ± 6.2%, respectively), when compared with the control cells stimulated with lipopolysaccharides (LPS) [82].

Species in the genus *Xenia* (family Xeniidae) (Figure 1B) are a rich source of diterpenoids. Xeniolides I, isolated from *Xenia novaebrittanniae* demonstrated antibacterial activity at a concentration of 1.25 mg/mL in *Escherichia coli* ATCC and *Bacillus subtilis* [85]. Blumiolide C, a diterpenoid from the *Xenia blumi* (presently accepted as *Xenia plicata*), exhibited potent cytotoxicity against mouse lymphocytic leukemia (P-388, ED_50_ 0.2 μg/mL) and human colon adenocarcinoma (HT-29, ED_50_ 0.5 μg/mL) cells [44].

Polyoxygenated cembranoids, crassocolides H–M from *Sarcophyton crassocaule*, demonstrated cytotoxicity against cancer cell lines of human medulloblastoma (Daoy cells) where crassocolides I and M were found to be more active (IC_50_ 0.8 and 1.1 μg/mL, respectively). Crassocolide H was also found to inhibit the growth of human oral epidermoid carcinoma (KB) cells (IC_50_ 5.3 μg/mL) and crassocolide L active against human cervical epitheloid carcinoma (HeLa) cells (IC_50_ 8.0 μg/mL) [62].

Another example of a potential new therapeutic anticancer agent is a cembranolide diterpene from *Lobophytum cristagalli*, which has shown a potent inhibitory activity (IC_50_ 0.15 μM) [59] over farnesyl protein transferase (FPT, an important protein in signal transduction and regulation of cell differentiation and proliferation [99]). This type of FPT inhibition enhanced interest in this group of metabolites [86]. Other species of this genus also showed cembranolide diterpenes (lobophytene) with significant cytotoxic activity against human lung adenocarcinoma (A549) and human colon adenocarcinoma (HT-29) cell lines [56]. *Lobophytum durum* and *Lobophytum crassum* produce durumolides A–C [60], durumhemiketalolide A–C [61] and crassumolides A and C [58], with anti-inflammatory effects. They have been shown to inhibit up-regulation of the pro-inflammatory iNOS and COX-2 proteins in LPS-stimulated murine macrophage cells at IC_50_ < 10 μM [58,60]. The diterpenoids, lobohedleolide, (7*Z)*-lobohedleolide, and 17-dimethylaminolobohedleolide, were isolated from the aqueous extract of *Lobophytum* species and exhibited moderate HIV-inhibitory activity (IC_50_ approximately 7–10 μg/mL) in a cell-based *in vitro* anti-HIV assay [57]. *Klyxum simplex* produces diterpene compounds, such as simplexin E, which at a concentration of 10 μM was found to considerably reduce the levels of iNOS and COX-2 proteins to 4.8 ± 1.8% and 37.7 ± 4.7%, respectively. These results have shown that this compound significantly inhibits the accumulation of the pro-inflammatory iNOS and COX-2 proteins in LPS-stimulated RAW264.7 macrophage cells [54]. This species also produces two diterpene compounds, klysimplexins B and H, exhibiting moderate cytotoxicity towards human carcinoma cell lines. Klysimplexin B exhibits cytotoxicity toward human hepatocellular carcinoma (Hep G2 and Hep 3B), human breast carcinoma (MDA-MB-231 and MCF-7), human lung carcinoma (A549) and human gingival carcinoma (Ca9-22) cell lines with IC_50_’s of 3.0, 3.6, 6.9, 3.0, 2.0, and 1.8 μg/mL, respectively. Metabolite klysimplexin H demonstrated cytotoxicity (IC_50_’s 5.6, 6.9, 4.4, 5.6, 2.8 and 6.1 μg/mL) toward human hepatocellular carcinoma (Hep G2 and Hep 3B), human breast carcinoma (MDA-MB-231 and MCF-7), human lung carcinoma (A549) and human gingival carcinoma (Ca9-22) cell lines, respectively [55].

In *Sinularia* sp. (Figure 1A), a tetraprenylated spermine derivative has been isolated—sinulamide— which revealed an H,K-ATPase inhibitory activity. H,K-ATPase is a gastric proton pump of stomach and is the enzyme primarily responsible for the acidification of the stomach contents. Its inhibition is a very common clinical intervention used in diseases including dyspepsia, peptic ulcer, and gastroesophageal reflux (GORD/GERD). Sinulide is a potential antiulcer drug, as it inhibits production of gastric acid by H,K-ATPase (IC_50_ 5.5 μM) [63]. Although it has been synthesized [100], no clinical trials seem to have been reported. The steroid gibberoketosterol [67], isolated from *Sinularia gibberosa*, and the diterpenoid querciformolide C [68] from *Sinularia querciformis*, showed significant inhibition of the up-regulation of the pro-inflammatory iNOS and COX-2 proteins in LPS-stimulated murine macrophages at concentration <10 μM [67,68]. *Paralemnalia thyrsoides* showed significant inhibition of pro-inflammatory iNOS protein expression (70% at IC_50_ 10 μM) [101]. *Sinularia* species produce significant molecules: lipids from *Sinularia grandilobata* and another unspecified species of *Sinularia* possesses antibacterial and antifungal activity [64]. The diterpene 11-episinulariolide from *Sinularia flexibilis* is an interesting antifoulant exhibiting strong algacidal properties [66]. This species also produces cembrenoids, named flexilarins, which evidence cytotoxic activity in cancer cell lines. Flexilarin D exhibited potent cytotoxicity in human hepatocarcinoma (Hep2) cells with IC_50_ 0.07 μg/mL, and moderate cytotoxic activity against human cervical epitheloid carcinoma (HeLa, IC_50_ 0.41 μg/mL), human medulloblastoma (Daoy, 1.24 μg/mL) and human breast carcinoma (MCF-7, 1.24 μg/mL) cell lines [65].

Antifouling agents from natural sources are of increasing interest since the International Maritime Organization (IMO) banned the use of certain antifouling agents, such as tri-*n*-butyltin (TBT), due to the ecological impacts of these biocides in the marine environment. Several studies have demonstrated that soft corals can yield large quantities of promising antifouling metabolites [102,103]. In fact, 17.95% of potential antifouling natural compounds are from cnidarians (e.g., soft coral) [104]. One of the most promising natural antifouling agent identified so far is an isogosterone isolated from an unspecified *Dendronephthya* [77].

The genus *Clavularia* contains secondary metabolites with unique structures and remarkable biological activities. Some of the species in this genus produce prostanoids (icosanoids) [45,72,73,105,106], steroids [75] and diterpenoids [70,107]. The bioactive marine diterpene, stolonidiol, isolated from an unidentified *Clavularia*, showed potent choline acetyltransferase (ChAT) inducible activity in primary cultured basal forebrain cells and clonal septal SN49 cells, suggesting that it may act as a potent neurotrophic factor-like agent on the cholinergic nervous system [69]. Cholinergic neurons in the basal forebrain innervate the cortex and hippocampus, and their function may be closely related to cognitive function and memory. The degeneration of neuronal cells in this brain region is considered to be responsible for several types of dementia including Alzheimer’s disease. One of the neurotransmitters, acetylcholine, is synthesized from acetyl coenzyme A and choline by the action of ChAT. Therefore, induction of ChAT activity in cholinergic neurons may improve the cognitive function in diseases exhibiting cholinergic deficits [108–110].

Prostanoids (claviridic acid) isolated from *Clavularia viridis* exhibited potent inhibitory effects on phytohemagglutinin-induced proliferation of peripheral blood mononuclear cells (PBMC, 5 μg/mL), as well as significant cytotoxic activity against human gastric cancer cells (AGS, IC_50_ 1.73–7.78 μg/mL) [71]. Claviridenone extracts also showed potent cytotoxicity against mouse lymphocytic leukemia (P-388) and human colon adenocarcinoma (HT-29), and exceptionally potent cytotoxicty against human lung adenocarcinoma (A549) cells, with ED_50_ between 0.52 pg/mL and 1.22 μg/mL [45]. Halogenated prostanoids also showed cytotoxic activity against human T lymphocyte leukemia cells (MOLT-4, IC_50_ 0.52 μg/mL), human colorectal adenocarcinoma (DLD-1, IC_50_ 0.6 μg/mL) and human diploid lung fibroblast (IMR-90, IC_50_ 4.5 μg/mL) cells [72]. The cyclopentenone prostanoid, bromovulone III-a promising marine natural compound for treatment of prostate, colon and hepatocellular carcinoma-showed anti-tumor activity against human prostate (PC-3) and human colon (HT29) cancer cells at an IC_50_ of 0.5 μM [73], and induced apoptotic signaling in a sequential manner in Hep3B cells [74]. In the case of prostate cancer cells, this compound displayed an anti-tumor activity 30 to 100 times more effective than cyclopentenone prostaglandins (known to suppress tumor cell growth and to induce apoptosis in prostate cancer cells), by causing a rapid redistribution and clustering of Fas (member of the tumor necrosis factor (TNF) receptor superfamily). Apoptotic stimulation of Fas by specific ligand or antibodies causes the formation of a membrane-associated complex comprising Fas clustering) in PC-3 cells [111]. *C. viridis* also produces steroids that show cytotoxic activity against human colorectal adenocarcinoma (DLD-1, 0.02 < IC_50_ < 50 μg/mL) and also against human T lymphocyte leukemia cells (MOLT-4, 0.01 < IC_50_ < 10 μg/mL), in the case of yonarasterols [75]. Stoloniferone additionally displayed potent cytotoxicity against mouse lymphocytic leukemia (P-388), human colon adenocarcinoma (HT-29) and human lung adenocarcinoma (A549) cells [45]. This species produces several compounds with anti-tumor activity in different types of human tumors, although more *in vitro* studies are needed to determine which compound are potential anticancer agents. *Clavularia koellikeri* produces diterpenoids as secondary metabolites, which display cytotoxic activity against human colorectal adenocarcinoma (DLD-1, IC_50_ 4.2 μg/mL) and strong growth inhibition against human T lymphocyte leukemia cells (MOLT-4, IC_50_ 0.9 μg/mL) [70].

In the genus *Cespitularia*, several interesting diterpenes of cembrane and neodolabellane skeletons have been identified. In *Cespitularia hypotentaculata* (family Xeniidae) a significant production of diterpenoids was detected. Cespitularin C exhibited potent cytotoxicity against mouse lymphocytic leukemia (P-388, ED_50_ 0.01 μg/mL) and human lung adenocarcinoma (A549, ED_50_ 0.12 μg/mL) cells, while cespitularin E exhibited potent cytotoxicity against human lung adenocarcinoma (A549, ED_50_ 0.034 μg/mL) cell cultures [84]. A less active diterpene, Asterolaurin A, from *Asterospicularia laurae* (a species from the same family) exhibited cytotoxicity against human hepatocellular carcinoma (HepG2) cells with an IC_50_ 8.9 μM [83].

*Telesto riisei* produces punaglandins, highly functional cyclopentadienone and cyclopentenone prostaglandins. Cyclopentenone prostaglandins have unique antineoplastic activity and are potent growth inhibitors in a variety of cultured cells. These punaglandins have been shown to inhibit P53 accumulation (a tumor suppressor protein) and ubiquitin isopeptidase activity (IC_50_ between 0.04 and 0.37 μM) (enzyme involved in protein degradation system) *in vitro* and *in vivo* [76]. Since these proteasome inhibitors exhibit higher antiproliferative effects than other prostaglandins [112], they may represent a new class of potent cancer therapeutics.

### 3.2. Order Gorgonacea (Sea Fans)

Gorgonians are a well-known source of compounds exhibiting significant biological activity [113]. Table 3 summarizes the most promising compounds from order Gorgonacea (class Anthozoa) described in the present review. Studies on *Isis hippuris* have resulted in the isolation of a series of novel metabolites such as sesquiterpenes [114], steroids [115], *A*-nor-hippuristanol [116] and isishippuric acid B [116]. These compounds exhibit potent cytotoxicity against cancer cell lines of human hepatocellular carcinoma (HepG2 and Hep3B, IC_50_ 0.08–4.64 μg/mL and 0.10–1.46 μg/mL, respectively) [116,117], human breast carcinoma (MCF-7, IC_50_ 0.20–4.54 μg/mL and MDA-MB-231, IC_50_ 0.13–2.64 μg/mL) [117], mouse lymphocytic leukemia (P-388), human lung adenocarcinoma (A549), and human colon adenocarcinoma (HT-29) with ED_50_ values less than 0.1 μg/mL [115,116] and IC_50_ of 0.1 μg/mL [114].

Species from the genus *Pseudopterogorgia* are a rich source of unusual biologically active diterpenoids, sesquiterpenes, and polyhydroxylated steroids, which exhibit diverse structures [127,140,141]. A sample of the organic extract of *Pseudopterogorgia bipinnata* was included in an initial screening carried out as part of an effort in the discovery of new antimalarial agents. This extract was found to be active in inhibiting the growth of *Plasmodium falciparum* (a protozoan parasite responsible for the most severe forms of malaria). Caucanolide A and D demonstrated significant *in vitro* antiplasmodial activity against chloroquine-resistant *P. falciparum* W2 (IC_50_ 17 μg/mL and IC_50_ 15 μg/mL, respectively) [128]. Three secosterols isolated from an unidentified gorgonian from genus *Pseudopterogorgia* inhibited human protein kinase C (PKC) α, βI, βII, γ, δ, ɛ, η, and ζ, with IC_50_ values in the range 12–50 μM [125]. PKC is a key player in cellular signal transduction and has been implicated in cancer, cardiovascular and renal disorders, immunosuppression, and autoimmune diseases such as rheumatoid arthritis [99]. Semisynthetic derivatives also showed a similar activity [125]. Promising antimicrobial substances were also reported from *Pseudopterogorgia rigida* (e.g., curcuphenol) [136] and from *Pseudopterogorgia elisabethae* (e.g., pseudopterosin X and Y) [129]. Ileabethoxazole, homopseudopteroxazole, caribenols A and B and elisapterosin B from *P. elisabethae* and bipinnapterolide B from *P. bipinnata* inhibit *Mycobacterium tuberculosis* H_37_Rv at a concentration of 12.5 μg/mL [131,133] (for elisapterosin B and homopseudopteroxazole) and at a concentration range of 128–64 μg/mL [130,132,142] (for others compounds). In fact, the inhibition of *M. tuberculosis* H_37_Rv is within the ranges recorded for rifampin [130]. *P. elisabethae* and *P. bipinnata* also produce antituberculosis compounds. Bielschowskysin, a naturally occurring diterpene isolated from *Pseudopterogorgia kallos* [135] and aberrarone isolated from *P. elisabethae* [134] exhibited antiplasmodial activity (IC_50_ 10 μg/mL) when tested against *P. falciparum*. The first compound was also found to display strong and specific *in vitro* cytotoxicity against the EKVX non-small cell lung cancer (GI_50_ < 0.01 μM) and CAKI-1 renal cancer (GI_50_ 0.51 μM) [135]. Bis(pseudopterane) amine from *Pseudopterogorgia acerosa* was found to exhibit selective activity against HCT116 (IC_50_ 4 μM) cell lines [126].

Fuscosides, originally isolated from *Eunicea fusca* [138], selectively and irreversibly inhibited leukotriene synthesis. Leukotrienes are molecules of the immune system that contribute to inflammation in asthma and allergic rhinitis and its production is usually related to histamine release [143]. Pharmacological studies indicated that fuscoside B inhibits the conversion of arachidonic acid (AA) to leukotriene B_4_ and C_4_ (LTB_4_ and LTC_4_) [138,144] by inhibiting the 5-Lipoxygenase (5-LO), in the case of LTB_4_ with an IC_50_ of 18 μM [144]. These selective inhibitors of lipoxygenase isoforms can be useful as pharmacological agents, as nutraceuticals or as molecular tools [99]. Sesquiterpenoids metabolites isolated from *Eunicea* sp. display antiplasmodial activity against the malaria parasite *P. falciparum* W2 (chloroquine-resistant) strain, with IC_50_ values ranging from 10 to 18 μg/mL [137].

The gorgonian *Junceella fragilis* produces secondary metabolites, frajunolides B and C, with anti-inflammatory effects towards superoxide anion generation and elastase release by human neutrophils, with an IC_50_ > 10 μg/mL [121]. When properly stimulated, activated neutrophils secrete a series of cytotoxins, such as the superoxide anion (O_2_^·−^), a precursor of other reactive oxygen species (ROS), granule proteases, and bioactive lipids [145,146]. The production of the superoxide anion is linked to the killing of invading microorganisms, but it can also directly or indirectly damage surrounding tissues. On the other hand, neutrophil elastase is a major secreted product of stimulated neutrophils and a major contributor to the destruction of tissue in chronic inflammatory disease [147]. The anti-inflammatory butenolide lipide [148] from the gorgonian *Euplexaura flava* [139] can be currently synthesized, opening the possibility of advancing into a new level of anti-inflammatory pharmaceuticals.

Some of the most interesting compounds identified so far in the on-going search for new anti-fouling agents have been recorded in the order Gorgonacea. Good examples of such compounds are juncin ZII from *Junceella juncea* [122], homarine from *Leptogorgia virgulata* and *Leptogorgia setacea* [123], pukalide and epoxypukalide recorded so far only from *L. virgulata* [124].

Species of genus *Briareum* (family Briareidae) (Figure 1D) (which commonly exhibit an incrusting appearance rather than the fan-like shape of many gorgonians) are widely abundant in Indo-Pacific and Caribean coral reefs. These organisms have been recognized as a valuable source of bioactive compounds with novel structural features. Briarane-related natural products are a good example of such promising compounds due to their structural complexity and biological activity [149,150]. Briaexcavatin E, from *Briareum excavata* (Nutting 1911), also occasionally referred to as *Briarium excavatum*, inhibited human neutrophil elastase (HNE) release with an IC_50_ between 5 and 10 μM [118]. Briaexcavatolides L and P, diterpenoids from the same species exhibited significant cytotoxicity against mouse lymphocytic leukemia (P-388) tumor cells with ED_50_ of 0.5 [119] and 0.9 μg/mL [151], respectively. Diterpenoids produced from *Briareum polyanthes* (presently accepted as *Briareum asbestinum*), namely Briarellin D, K and L, exhibited antimalarial activity against *P. falciparum* with an IC_50_ between 9 and 15 μg/mL [120].

### 3.3. Other Orders

Sea anemones (order Actiniaria) are a rich source of biologically-active proteins and polypeptides. Several cytolytic toxins, neuropeptides and protease inhibitors have been identified from them [48]. In addition to several equinatoxins, potent cytolytic proteins and an inhibitor of papain-like cysteine proteinases (equistatin), were isolated from the sea anemone *Actinia equina* [152]. Equistatin has been shown to be a very potent inhibitor of papain and a specific inhibitor of the aspartic proteinase cathepsin D [153]. While papain-like cysteine proteases have been implicated in various diseases of the central nervous system, such as brain tumors, Alzheimer’s disease, stroke, cerebral lesions, neurological autoimmune diseases and certain forms of epilepsy [154], aspartic proteinase cathepsin D is involved in the pathogenesis of breast cancer [155] and possibly Alzheimer’s disease [156].

Cycloaplysinopsin C, a bis(indole) alkaloid isolated from *Tubastrea* sp. (order Scleractinia), was found to inhibit growth of two strains of *P. falciparum*, one chloroquine-sensitive (F32/Tanzania) and other chloroquine-resistant (FcB1/Colombia) with IC_50_ 1.48 and 1.2 μg/mL, respectively [51]. Cladocorans A and B, isolated from *Cladocora caespitosa* (order Scleractinia) [49], are marine sesterterpenoids which possess a γ-hydroxybutenolide moiety, which is thought to be responsible for the biological activity of these compounds. The potent anti-inflammatory activity of these natural metabolites was attributed to the inhibition of secretory phospholipase A_2_ (sPLA_2_, IC_50_ 0.8–1.9 μM). Given the general role of inflammation in diseases that include bronchial asthma and rheumatoid arthritis, identifying and developing potent inhibitors of sPLA2 continues to be of great importance for the pharmaceutical industry, with this type of metabolite being of paramount importance for future research [50].

## 4. Class Hydrozoa

Class Hydrozoa includes seven orders and nearly 3500 valid species (Table 1), some of which are solitary, some of which are colonial. Among the most emblematic species are probably hydroids and the Portuguese man-o-war (*Physalia physalis*). Despite the large number of species in class Hydrozoa, only a few of them have yielded interesting MNPs in the last decade.

Immune escape plays an important role in cancer progression and, although not completely understood, it has been proposed that indoleamine 2,3-dioxygenase (IDO) plays a central role in evasion of T-cell-mediated immune rejection [157]. IDO catalyzes the oxidative cleavage of the 2,3 bond of tryptophan, which is the first and rate-limiting step in the kynurenine pathway of tryptophan catabolism in mammalian cells [158]. The polyketides annulins A, B, and C, purified from the marine hydroid *Garveia annulata* (order Anthoathecata), potently inhibited IDO *in vitro* (*K_i_* 0.12–0.69 μM) [159]. These annulins are more powerful than most tryptophan analogues known to be IDO inhibitors. These compounds are active at concentrations higher than ~10 μM and therefore more effective than 1-methyltryptophan (*K_i_* 6.6 μM), one of the most potent IDO inhibitors currently available [160]. Solandelactones C, D, and G are cyclopropyl oxylipins isolated from the hydroid *Solanderia secunda* (order Anthoathecata) and exhibit moderate inhibitory activity against farnesyl protein transferase (FPT, 69, 89, and 61% inhibition, respectively) at a concentration of 100 μg/mL [161]. Note that FPT is associated with cell differentiation and proliferation and its inhibition may be a target for novel anticancer agents (as already referred above for the soft coral *L. cristagalli*).

## 5. Class Scyphozoa

Approximately 200 species are currently classified in three orders in class Scyphozoa (Table 1). However, in the last decade, only a single MNP purified from the mesoglea of the jellyfish *Aurelia aurita* (order Semaeostomeae) was considered to be promising enough to be included in the present work. This compound is a novel endogenous antibacterial peptide, aurelin, which exhibited activity against Gram-positive and Gram-negative bacteria. As an example, aurelin displayed an IC_50_ of 7.7 μg/mL for *Esherichia coli* (Gram negative bacteria) [162].

## 6. Other Classes

The classes Staurozoa, Cubozoa and Polypodiozoa are the least speciose in the phylum Cnidaria (Table 1). This fact may explain the current lack of data on secondary metabolites produced by these organisms. It is possible that with growing bioprospecting new MNPs may be revealed once these cnidarian species are screened. Cubozoa (box jellies), for example, produce some of the most harmful cnidarian toxins for humans [163].

## 7. Exploring the Unexplored and Being Creative: Future Perspectives for the Bioprospecting of Cnidarians

For several years, the bioprospecting of cnidarians was commonly limited to habitats that could be readily sampled by researchers, such as shallow coral reefs and the intertidal region. However, with improvements in SCUBA gear, researchers are now able to dive deeper and longer, allowing them to collect a wider range of cnidarian species for the screening of MNPs. The growing efforts to explore Earth’s last frontier, the deep sea, made it possible to start bioprospecting several unique marine ecosystems that had remained either previously unrecorded or inaccessible to researchers [164]. New cnidarian species (some of them belonging to new genera and probably even to new families) (e.g., [165,166]) are currently being sampled from the deep sea. These findings suggest that many new species are yet to be discovered along deep continental margins [167] and open good perspectives for the discovery of new MNPs with ongoing surveys of deep sea fauna. Cnidarians are known to colonize unique deep sea biotopes, namely chemosynthetic sites (such as hydrothermal vents, cold seeps and whale falls [168]), as well as seamounts [169]. Some of these organisms are endemic to these habitats and display remarkable adaptations to extreme environments (e.g., chemosynthetic sea anemones) [170]. These species are certainly interesting candidates for the discovery of new MNPs [171]. However, some of these remarkable biotopes, namely deep sea coral reefs, are already facing serious threats to their conservation [169] and thus, the bioprospecting of these and other endangered habitats must be carefully addressed [164,172].

Another interesting source of cnidarian species for bioprospecting is the marine aquarium industry. Over 200 species of hard and soft corals, along with several other anemone, zoanthid and corallimorph species, are harvested every year from coral reefs to supply the marine aquarium trade [173]. However, researchers using these organisms in the bioprospecting of new MNPs must be aware that it is not commonly possible to get reliable information on either the place of origin or the scientific name of most traded specimens. With the advent of high-throughput screening (HTS) [174], it will be possible to rapidly survey these organisms for interesting MNPs, although HTS of natural sources may present several challenges (see [175,176]). If necessary, additional biomass of target organisms producing interesting MNPs can be achieved using inexpensive techniques [177,178] and eliminate problems commonly faced by researchers screening marine organisms for MNPs–the loss of the source and reproducibility [176].

The discovery of a new compound commonly requires only small amounts of biomass. However the production of these compounds at a scale large enough to fulfill commercial applications is still nearly impossible [179]. In theory, large-scale production of bioactive compounds can be achieved by chemical synthesis or through extraction from marine animals, either harvested from the sea or maricultured. The existence of ecophysiological diversity (e.g., differences between individuals often due to differences in environmental interactions) can interfere with the production of MNPs and must be carefully addressed in future efforts for large-scale production of these compounds. The harvest of target animals from the wild for the production of chemical compounds is commonly an unsustainable solution, while mariculture has proven to be more technically challenging and expensive than previously assumed [180]. In other considerations, chemical synthesis is not yet developed to synthesize complex molecules at the kilogram scale and, in cases where this may already be technically possible, most of the compounds cannot be synthesized at a price affordable for commercial applications [179]. Potential solutions for such bottlenecks may be the use of diverted total synthesis [181] and/or metabolic engineering [182].

There is growing evidence that microbes associated with marine invertebrates may be the true producers of some of the bioactive compounds isolated from these animals [179]. Whether this is the case of bioactive compounds currently assumed to be produced by cnidarians remains unanswered [183,184]. If so, we face another constraint for the commercial use of these compounds, as the culture of symbiotic microorganisms is generally not possible using classic/standardized methodologies.

## 8. Conclusions

The intense pressure to find and develop more profitable molecules for all sorts of industries continues to fuel the bioprospecting of marine invertebrates. Although the phylum Cnidaria is not the most significantly bioprospected at present, this review shows that some cnidarian species are promising sources of marine bioactive compounds of medical, economic and scientific interest. Green fluorescent protein (GFP), GPF-like proteins, red fluorescent and orange fluorescent protein (OPF) are good examples of biotechnological metabolites currently employed as molecular biomarkers. They were first purified from a fluorescent hydrozoan medusa [185] and since then have been recorded in other cnidarian species [186–191].

**Figure 2:**
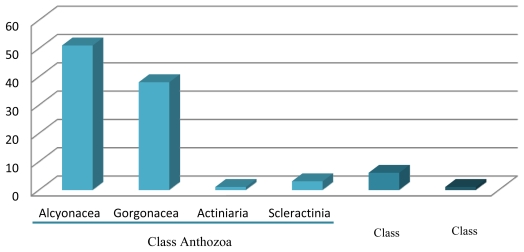
Marine bioactive compounds with high biotechnological potential studied from the phylum Cnidaria in the last decade.

In the present survey, only about 0.31% of extant cnidarian species are represented, with class Anthozoa displaying by far the highest number of promising MNPs (Figure 2). This result is probably due to the fact that this class is the most speciose in the phylum (Table 1). Additionally, many anthozoans occupy marine habitats which can be readily accessed for the collection of biomass (e.g., coral reefs and intertidal regions), which facilitates bioprospecting. Of all the compounds presented in this review, 84% were detected in cnidarians collected from tropical waters (mostly from Southeast Asia and the Caribbean Sea) and the remaining 16% were recorded from species mostly occupying temperate waters (e.g., European countries and Japan).

**Figure 3:**
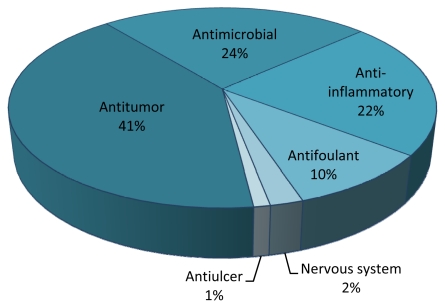
Distribution in drug classes of marine bioactive compounds with high biotechnological potential studied from cnidarian species in the last decade.

**Figure 4:**
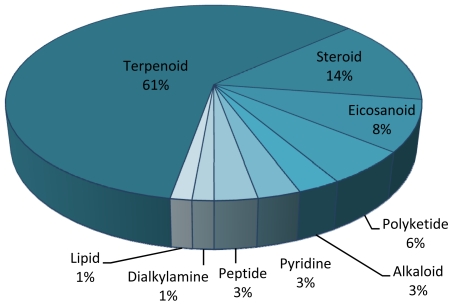
Distribution of chemistry classes of marine bioactive compounds with high biotechnological potential studied from cnidarian species in the last decade.

Antitumor drugs are the main area of interest in the screening of MNPs from cnidarians (41%, Figure 3). This is not surprising, as the major financial effort for the screening of new marine compounds is made in cancer research [192]. Terpenoids (terpenoid, diterpenoid, sesquiterpenoid, sesterterpenoid, cembranoid) [193] (Figure 4) are the main chemistry group within the MNPs analyzed in this survey.

Even though most pharmaceutical industries abandoned their natural product-based discovery programs over a decade ago, the lack of new compounds in their pipelines in some strategic areas (e.g., antibiotics) suggests that renewed interest in this field is imminent. The establishment of small biotech companies can play a decisive role in the initial discovery of promising marine bioactive compounds, as these enterprises will work closely together with academics and governmental agencies performing the initial steps in the discovery of new MNPs. Collaboration between private companies and public institutions can be of paramount importance for financial support in the discovery process. On the other side, crude extracts and pure compounds produced by academic laboratories may be screened by diverse bioassays as a part of broader collaboration programs, nationally and internationally, with private biotech companies. One challenge for universities is to devise mechanisms that protect intellectual property and simultaneously encourage partnerships with the private sector, by recognizing that the chances of a major commercial pay-off are small if drug discovery is pursued by a single institution [3].

The commercial use of some promising marine bioactive compounds isolated from cnidarians may be several years away. New compounds other than toxins and venoms produced by members of this highly diverse group of marine invertebrates may be discovered in the quest for new marine products.

## Figures and Tables

**Table 1:** Classes and orders in the phylum Cnidaria followed in this paper.

Phylum	Class	Order	
Cnidaria (≈11,287 species)	Anthozoa (≈7500 species)	Actiniaria Antipatharia Ceriantharia Corallimorpharia Scleractinia	Zoanthidea Alcyonacea Gorgonacea Helioporacea Pennatulacea
	Cubozoa (≈36 species)	Carybdeida	Chirodropida
	Hydrozoa (≈3500 species)	Anthoathecata Leptothecata Siphonophorae Actinulida	Limnomedusae Narcomedusae Trachymedusae
	Polypodiozoa (1 species)		
	Scyphozoa (≈200 species)	Coronatae Rhizostomeae	Semaeostomeae
	Staurozoa (≈50 species)	Stauromedusae	

**Table 2:** Most promising compounds studied in the last decade from cnidarian species in order Alcyonacea (soft corals), class Anthozoa.

Family and Species	Drug Class	Compound	Chemistry	Country	Ref.
Alcyoniidae					
*Klyxum simplex*	Anti-inflammatory	Simplexin E	Diterpenoid	TAIW	[54]
*Klyxum simplex*	Antitumor	Klysimplexin B and H	Diterpenoid	TAIW	[55]
*Lobophytum* sp.	Antitumor	Lobophytene	Diterpenoid	VN	[56]
*Lobophytum* sp.	Anti-HIV	Lobohedleolide	Diterpenoid	PHL	[57]
*Lobophytum* sp.	Anti-HIV	(7*Z*)-lobohedleolide,	Diterpenoid	PHL	[57]
*Lobophytum* sp.	Anti-HIV	17-dimethylamino lobohedleolide	Diterpenoid	PHL	[57]
*Lobophytum crassum*	Anti-inflammatory	Crassumolides A and C	Terpenoid	TAIW	[58]
*Lobophytum cristagalli*	Antitumor	Cembranolide diterpene	Diterpenoid	RSC	[59]
*Lobophytum durum*	Anti-inflammatory	Durumolides A–C	Terpenoid	TAIW	[60]
*Lobophytum durum*	Anti-inflammatory	Durumhemiketalolide A–C	Cembranoid	TAIW	[61]
*Sarcophyton crassocaule*	Antitumor	Crassocolides H–M	Cembranoid	TAIW	[62]
*Sinularia* sp.	Antiulcer	Sinulide	Spermine		[63]
*Sinularia* sp.	Antimicrobial	Lipids	Polyketide	RUS	[64]
*Sinularia flexibilis*	Antitumor	Flexilarin D	Cembranoid	TAIW	[65]
*Sinularia flexibilis*	Antifoulant	11-episinulariolide	Diterpenoid	AUS	[66]
*Sinularia gibberosa*	Anti-inflammatory	Gibberoketosterol	Steroid	TAIW	[67]
*Sinularia querciformis*	Anti-inflammatory	Querciformolide C	Terpenoid	TAIW	[68]
Clavulariidae					
*Clavularia* sp.	Nervous system	Stolonidiol	Diterpenoid	JPN	[69]
*Clavularia koellikeri*	Antitumor	Cembrane-type diterpenoid	Diterpenoid	JPN	[70]
*Clavularia viridis*	Antitumor	Claviridic acid	Prostanoid	TAIW	[71]
*Clavularia viridis*	Antitumor	Clavulones	Prostanoid	TAIW	[71]
*Clavularia viridis*	Antitumor	Claviridenone	Prostanoid	TAIW	[45]
*Clavularia viridis*	Antitumor	Halogenated prostanoids	Prostanoid	JPN	[72]
*Clavularia viridis*	Antitumor	Bromovulone III	Prostanoid	TAIW	[73,74]
*Clavularia viridis*	Antitumor	Yonarasterols	Steroid	JPN	[75]
*Clavularia viridis*	Antitumor	Stoloniferone E	Steroid	TAIW	[45]
*Telesto riisei*	Antitumor	Punaglandins	Prostaglandin	USA	[76]
Nephtheidae					
*Dendronephthya* sp.	Antifoulant	Isogosterones A–D	Steroid	JPN	[77]
*Dendronephthya rubeola*	Antitumour	Capnell-9(12)-ene-8β,10α-diol	Sesquiterpenoid	DE	[78,79,80]
*Nephthea chabroli*	Antitumor	Chabranol	Terpenoid	TAIW	[81]
*Nephthea erecta*	Anti-inflammatory	Ergostanoids 1 and 3	Ergostanoid	TAIW	[82]
Xeniidae					
*Asterospicularia laurae*	Antitumor	Asterolaurin A	Diterpenoid	TAIW	[83]
*Cespitularia hypotentaculata*	Antitumor	Cespitularin C	Diterpenoid	TAIW	[84]
*Xenia novaebritanniae*	Antibacterial	Xeniolide I	Diterpenoid	ISR	[85]
*Xenia plicata*	Antitumor	Blumiolide C	Diterpenoid	TAIW	[44]

AUS: Australia; DE: Germany; ISR: Israel; JPN: Japan; PHL: Philippines; RSC: Republic of Seychelles; RUS: Russia; TAIW: Taiwan; VN: Vietnam.

**Table 3:** Most promising compounds studied in the last decade from cnidarian species in order Gorgonacea (sea fans), class Anthozoa.

Family and Species	Drug Class	Compound	Chemistry	Country	Ref.
Briareidae					
*Briareum excavate*	Anti-inflammatory	Briaexcavatin E	Diterpenoid	TAIW	[118]
*Briareum excavate*	Antitumor	Briaexcavatolides L and P	Diterpenoid	TAIW	[119]
*Briareum asbestinum*	Antimalarial	Briarellin D, K and L	Diterpenoid	PAN, USA	[120]
Ellisellidae					
*Junceella fragilis*	Anti-inflammatory	Frajunolides B and C	Terpenoid	TAIW	[121]
*Junceella juncea*	Antifoulant	Juncin ZII	Diterpenoid	TAIW	[122]
Gorgoniidae					
*Leptogorgia setácea*	Antifoulant	Homarine	Pyridine	GEO	[123]
*Leptogorgia virgulata*	Antifoulant	Homarine	Pyridine	GEO	[123]
*Leptogorgia virgulata*	Antifoulant	Pukalide	Diterpenoid	USA	[124]
*Leptogorgia virgulata*	Antifoulant	Epoxypukalide	Diterpenoid	USA	[124]
*Pseudopterogorgia* sp.	Antitumor	Secosterols	Sterol	USA	[125]
*Pseudopterogorgia* sp.	Anti-inflammatory	Secosterols	Sterol	USA	[125]
*Pseudopterogorgia acerosa*	Antitumor	Bis(pseudopterane) amine	Dialkylamine	BHS	[126]
*Pseudopterogorgia bipinnata*	Antituberculosis	Bipinnapterolide B	Terpenoid	USA	[127]
*Pseudopterogorgia bipinnata*	Antimalarial	Caucanolide A and D	Diterpenoid	COL, PAN, USA	[128]
*Pseudopterogorgia elisabethae*	Antimicrobial	Pseudopterosin X	Diterpenoid	USA	[129]
*Pseudopterogorgia elisabethae*	Antituberculosis	Ileabethoxazole	Diterpenoid	USA	[130]
*Pseudopterogorgia elisabethae*	Antituberculosis	Homopseudopteroxazole	Diterpenoid	USA	[131]
*Pseudopterogorgia elisabethae*	Antituberculosis	Caribenols A and B	Terpenoid	USA	[132]
*Pseudopterogorgia elisabethae*	Antituberculosis	Elisapterosin B	Diterpenoid	USA	[133]
*Pseudopterogorgia elisabethae*	Antimalarial	Aberrarone	Diterpenoid	COL	[134]
*Pseudopterogorgia kallos*	Antimalarial	Bielschowskysin	Diterpenoid	PAN, USA	[135]
*Pseudopterogorgia kallos*	Antitumor	Bielschowskysin	Diterpenoid	PAN, USA	[135]
*Pseudopterogorgia rígida*	Antimicrobial	Curcuphenol	Terpenoid	USA	[136]
Isididae					
*Isis hippuris*	Antitumor	Suberosenol B	Terpenoid	TAIW	[114]
*Isis hippuris*	Antitumor	Polyoxygenated steroids	Steroid	IND	[115,117]
*Isis hippuris*	Antitumor	A –nor-hippuristanol	Steroid	TAIW	[116]
*Isis hippuris*	Antitumor	Isishippuric acid B	Steroid	TAIW	[116]
Plexauridae					
*Eunicea* sp.	Antimalarial	Sesquiterpenoids	Sesquiterpenoid	COL, PAN, USA	[137]
*Eunicea fusca*	Anti-inflammatory	Fuscisides	Diterpenoid	USA	[138]
*Euplexaura flava*	Anti-inflammatory	Butenolide	Lipid	JPN	[139]

ND: Not Determined; BHS: Bahamas; COL: Colombia; GEO: Georgia; IND: Indonesia; PAN: Panama; TAIW: Taiwan; USA: United States of America.
